# A Low-Computing-Complexity Touch Signal Detection Method and Analog Front-End Circuits Based on Cross-Correlation Technology for Large-Size Touch Panel

**DOI:** 10.3390/s22124354

**Published:** 2022-06-08

**Authors:** Xiaoyu Guo, Hongge Li, Yuhao Chen, Wei Sun

**Affiliations:** 1 School of Electronic and Information Engineering, Beihang University, Beijing 100191, China; eeguoxiaoyu@buaa.edu.cn (X.G.); zy1902608@buaa.edu.cn (Y.C.); 2 BOE Technology Group Co., Ltd., Beijing 100015, China; sunwei_boeot@boe.com.cn

**Keywords:** analog front-end (AFE), weak touch signal detection, cross-correlation, low computing complexity, large-size touch screen panel

## Abstract

This paper proposes a low-computing-complexity touch signal detection method and analog front-end (AFE) circuits based on cross-correlation technology for large mutual capacitance touch screen panels (TSPs). To solve the traditional touch signal detection method problem of lots of invalid data being sampled and processed in a large-size TSP, the proposed method only samples and processes the signals around the touch points rather than full-screen data to decrease the computing complexity and analog–digital convertor (ADC) acquisition number. Compared with the traditional method, the proposed touch points search algorithm complexity decreases from *MN* to *M* + *nN* where *M*, *N*, and *n* are the number of RX channels, TX channels, and touch points, respectively. The maximum ADC acquisition number of the proposed method decreases from *MN* to 18*n*. Based on the proposed touch signal detection method, the AFE circuits are designed by a 0.11 μm process. The proposed dual cross-correlation AFE achieves detection of the weak touch signal submerged in the large display panel noise. The average channel area and power consumption are decreased to 0.015 mm^2^ and 0.227 mW, respectively. The maximum frame rate is 384.6 Hz with 10 touch points. The proposed cross-correlation AFE achieves a high frame rate while reducing the die area and power consumption.

## 1. Introduction

Nowadays, mutual capacitance touch screen panels (TSPs) are widely used in people’s daily lives due to the advantages of mutual capacitance screens, such as a high light transmission rate, fast response time, long life, and multiple-touch points support [1,2]. The touch signals recognition algorithm and circuits are two essential parts of the mutual capacitance touch system that determine the touch signal recognition capability. The touch signal recognition algorithm cannot be separated from the support of the analog front-end (AFE) circuit. With an increase in TSP size, new challenges arise related to the design of the AFE circuit.

The first challenge is that a large amount of invalid data is sampled and processed for the large-size TSP. Compared with a small-size TSP, the proportion of the untouched area to the total panel area increases significantly for the large-size TSP. Most of the touch screen area is not touched in the large-size touch panel because human fingers occupy only a small area, and most of the data sampled by the analog-digital convertors (ADC) are invalid [3,4]. In other words, the percentage of invalid data sampled by ADC increases correspondingly. A low-computing-complexity acquisition method to solve this problem is proposed in this paper. This method first judges the coarse position of the touch points. Then, only the signals around the touch points are sampled instead of sampling the full-screen data to calculate the precise touch point coordinates. This acquisition method effectively reduces the data acquisition and the requirement of the ADC. Only a low-speed ADC is required to complete the acquisition of touch point peripheral signals. Moreover, the computing complexity of the digital back-end is reduced because of insufficient data.

The second challenge is that the detection of the touch signal becomes more difficult because of the smaller receiving signal and the larger interference noise for the large-size TSP. The touch screen gradually transitions from on-glass to on-cell and in-cell to thin the screen and reduce the cost of the development of the touch screen [1,5]. However, the space between the touch panel and the display driver circuit becomes smaller, the capacitance between the touch panel and the display driver circuit becomes smaller, and the display driver noise on the touch electrode becomes larger [6,7,8,9,10]. In large-size displays, the display drivers are high-voltage circuits, so the display drive noise on the touch electrode further increases, and the touch signal is submerged in the display driver noise [11,12,13]. In addition, the touch electrode becomes longer with the increase in the touch panel size, and the low-frequency external noise is coupled to the touch electrode more easily. Compared with that of the small-size TSP, the signal received in the large-size TSP is smaller due to the larger panel load [7]. Overall, the difficulty of touch signal detection increases due to the smaller receiving signal and the larger noise for the large TSP. In order to detect the weak touch signals submerged in the noise, this paper proposes a novel AFE circuit based on cross-correlation technology to improve the recognition ability of touch signals.

The third challenge is the tradeoff of the high frame rate and small die area for the large-size TSP. There are three main driving methods for mutual capacitive TSP, including the serial driving method, parallel coded driving method, and parallel multi-frequency driving method [14,15,16,17,18,19,20,21,22,23,24,25,26]. The number of electrodes increases with the increase in the mutual capacitance TSP size [27,28]. The serial driving method cannot achieve a high frame rate because the scan time of a frame increases dramatically. The frame rate is reduced due to the long coding sequence in the parallel coding driver method for the large-size TSP [29]. To realize a high frame rate for the large-size TSP, the multi-frequency driving method, which transmits different frequency signals to different transmit channels, is adopted. However, fast Fourier transform (FFT) circuits that occupy a sizeable area are required to distinguish different transmit channels [30,31,32]. The proposed multi-frequency driving AFE circuits distinguish different transmit channels by cross-correlation technology without a complex FFT module for the large-size mutual capacitance TSP to solve this problem. The proposed cross-correlation AFE achieves a high frame rate while reducing the die area and power consumption.

This paper proposes a low computing complexity touch signal detection method and AFE circuits based on cross-correlation technology for large-size mutual capacitance TSPs. The main contributions of this work are as follows:

1. A novel cross-correlation operation AFE architecture and analog cross-correlation computing circuits are proposed;
2. The proposed cross-correlation AFE method achieved the detection of the weak touch signal submerged in the large display panel noise;
3. The proposed cross-correlation multi-frequency driving AFE realizes a high frame rate without complex FFT circuits to reduce the die area and power consumption;
4. The proposed touch signal detection method only samples and processes the signals around the touch points rather than full-screen data to decrease the computing complexity and ADC acquisition number.

The remaining sessions of this paper are organized as follows: Section 2 introduces the technique of touch signal identification under noise drowning, including channel differencing and cross-correlation techniques. Section 3 presents the AFE architecture of the low-computing-complexity acquisition method based on cross-correlation technology. Then, the cross-correlation circuits are described in detail. Section 4 gives the results and a discussion of the proposed method and AFE circuits. Section 5 draws some conclusions.

## 2. Channel Difference and Cross-Correlation Analysis

### 2.1. Channel Difference Analysis

**Figure 1:**
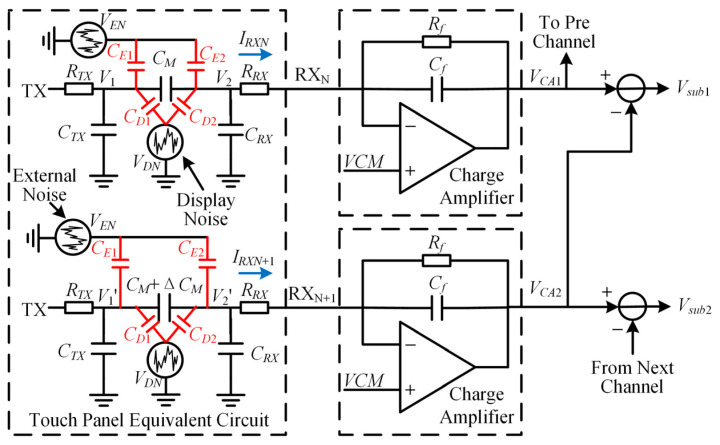
Two adjacent received channel differential operation circuits and touch panel equivalent circuits with noise sources.

The differential operation of two adjacent received channel signals can reduce the interference of common-mode noise and extracts the weak touch signal. Figure 1 shows the two adjacent received channel differential operation circuits and touch panel equivalent circuits with noise sources. *TX* is the transmitting signal. *RX_N_* and *RX_N_*_+1_ are two adjacent receiving signals. In the touch panel equivalent circuit, *C_TX_* and *C_RX_* are the TX electrode to ground capacitance and RX electrode to ground capacitance, respectively. *R_TX_* and *R_RX_* are the TX electrode equivalent resistance and RX electrode equivalent resistance, respectively. *C_M_* is the mutual capacitance between the TX electrode and the RX electrode. When there is a finger touch, *C_M_* changes to *C_M_* + Δ*C_M_*. *R_f_* and *C_f_* are the feedback resistance and feedback capacitance of the charge amplifier, respectively. The differential function is achieved by subtracting the charge amplifier outputs of two adjacent channels.

Consider display noise *V_DN_* and external noise *V_EN_*. *C_D_*_1_ is the equivalent capacitance between the display panel and the TX electrode. *C_D_*_2_ is the equivalent capacitance between the display panel and the RX electrode. *C_E_*_1_ is the equivalent capacitance between the external noise source and the TX electrode. *C_E_*_2_ is the equivalent capacitance between the external noise source and the RX electrode. Since the noise interference in the two adjacent received channels is basically the same, the differential operation could effectively eliminate the common noise interference.

According to the superposition theorem, the charge amplifier output voltage *V_CA_*_1_ and *V_CA_*_2_ are:

(1) $$V_{CA1}=H_{TX}(s)V_{TX}+H_{DN}(s)V_{DN}+H_{EN}(s)V_{EN}$$

(2) $$V_{CA2}={H_{TX}}^{\prime }(s)V_{TX}+{H_{DN}}^{\prime }(s)V_{DN}+{H_{EN}}^{\prime }(s)V_{EN},$$

where *H_TX_*(*s*), *H_DN_*(*s*), and *H_EN_*(*s*) are the transfer functions of *TX* signal, display noise, and external noise, respectively, if there is not a touch point. *H_TX_′*(*s*), *H_DN_′*(*s*), and *H_EN_′*(*s*) are the transfer functions of *TX* signal, display noise, and external noise, respectively, if there is a touch point.

The subtractor output voltage *V_sub_*_1_ is:

(3) $$V_{sub1}=V_{CA1}-V_{CA2}=\Delta H_{TX}(s)V_{TX}+\Delta H_{DN}(s)V_{DN}+\Delta H_{EN}(s)V_{EN}.$$

where Δ*H_TX_*(*s*), Δ*H_DN_*(*s*) and Δ*H_EN_*(*s*) are the difference of transfer functions. Δ*H_TX_*(*s*) = *H_TX_*(*s*) − *H_TX_′*(*s*); Δ*H_DN_*(*s*) = *H_DN_*(*s*) − *Hs_DN_′*(*s*); Δ*H_EN_*(*s*) = *H_EN_*(*s*) − *H_EN_′*(*s*).

To solve Δ*H_TX_*(*s*), set *V_DN_* and *V_EN_* to 0. In the touch panel equivalent circuit, *V*_1_ and *V*_2_ are two node voltages on both sides of the *C_M_*. According to Kirchhoff’s current law, we obtain

(4) $$\frac{V_{TX}-V_{1}}{R_{TX}}+\frac{V_{2}-V_{1}}{\frac{1}{sC_{M}}}=\frac{V_{1}}{\frac{1}{s(C_{TX}+C_{D1}+C_{E1})}}$$

(5) $$\frac{V_{1}-V_{2}}{\frac{1}{sC_{M}}}+\frac{VCM-V_{2}}{R_{RX}}=\frac{V_{2}}{\frac{1}{s(C_{RX}+C_{D2}+C_{E2})}},$$

where *VCM* is the common voltage. The voltage of both *RX_N_* and *RX_N_*_+1_ is *VCM*. Let *C* = *C_TX_* + *C_D_*_1_ + *C_E_*_1_ = *C_RX_* + *C_D_*_2_ + *C_E_*_2_. Because *C_M_* << *C_TX_* = *C_RX_*, *C_M_* << *C*. The node voltage *V*_2_ is:

(6) $$V_{2}=\frac{VCM}{sCR_{RX}+1}+\frac{sC_{M}R_{RX}V_{TX}}{\left(sCR_{TX}+1)(sCR_{RX}+1\right)}.$$

Similarly,

(7) $${V_{2}}^{\prime }=\frac{VCM}{sCR_{RX}+1}+\frac{s(C_{M}+\Delta C_{M})R_{RX}V_{TX}}{\left(sCR_{TX}+1)(sCR_{RX}+1\right)}.$$

The charge amplifier output voltage *V_CA_* is:

(8) $$V_{CA}=VCM-I_{RX}(R_{f}||\frac{1}{sC_{f}}).$$

where *I_RX_* is the current of RX.

The subtractor output voltage *V_sub_*_1_ is:

(9) $$V_{sub1}=V_{CA1}-V_{CA2}=(I_{RXN+1}-I_{RXN})(R_{f}||\frac{1}{sC_{f}})=\frac{{V_{2}}^{\prime }-V_{2}}{R_{RX}}(R_{f}||\frac{1}{sC_{f}}).$$

Substituting Equations (6) and (7) into Equation (9), we obtain

(10) $$V_{sub1}=\frac{sR_{f}\Delta C_{M}}{\left(sC_{f}R_{f}+1)(sCR_{TX}+1)(sCR_{RX}+1\right)}V_{TX}=\Delta H_{TX}(s)V_{TX},$$

where

(11) $$\Delta H_{TX}(s)=\frac{sR_{f}\Delta C_{M}}{\left(sC_{f}R_{f}+1)(sCR_{TX}+1)(sCR_{RX}+1\right)}.$$

If there is a touch point, the variation of *C_M_* is Δ*C_M_*. The subtractor output voltage signal does not equal zero. If there is not a touch point, the variation of Δ*C_M_* equals zero. The subtractor output voltage signal equals zero.

Similarly, Δ*H_DN_*(*s*) and Δ*H_EN_*(*s*) are functions of Δ*C_M_* where Δ*H_DN_*(*s*) = *f*(Δ*C_M_*) and Δ*H_EN_*(*s*) = *g*(Δ*C_M_*). If there is not a touch point, the variation of Δ*C_M_* equals zero. Δ*H_DN_*(*s*) and Δ*H_EN_*(*s*) are equal to zero. If there is a touch point, Δ*H_DN_*(*s*) and Δ*H_EN_*(*s*) do not equal zero. In other words, after the two channels are differenced, there is still residual display noise and external noise.

### 2.2. Cross-Correlation Analysis

Cross-correlation technology is an effective way to detect weak signals submerged in the noise. If there is a touch point near the touch coordinate (*p*, *q*), *p* is the horizontal ordinate of the RX channel, and *q* is the vertical ordinate of the TX channel. The transfer function *H_pq_*(*s*) becomes *H_pq_′*(*s*), where 1 ≤ *p* ≤ *M* and 1 ≤ *q* ≤ *N*.

The differential charge amplifier output signal *V_subi_*(*t*) of RX channel *i* is:

(12) $$V_{subi}(t)=\{\begin{array}{cc} \Delta H_{TX}(s)TX_{q}(t)+\Delta H_{DN}(s)V_{DN}+\Delta H_{EN}(s)V_{EN}, & i=p\\ 0, & i\neq p \end{array}.$$

There is a phase difference between the differential charge amplifier output signal and the *TX*(*t*). To avoid the result of cross-correlation operation between two signals with a zero frequency, two orthogonal transmitted signals, *x*(*t*) and *x’*(*t*), with the same frequency, are used for cross-correlation operations. The Fourier expansion form of the transmitted two orthogonal square waves with the same frequency is [33]:

(13) $$x(t)=TX_{q\_0^{\circ }}(t)=A\sum_{n=0}^{\infty }\frac{1}{2n+1}\sin(2n+1)\omega_{q}t$$

(14) $$x^{\prime }(t)=TX_{q\_90^{\circ }}(t)=A\sum_{n=0}^{\infty }\frac{1}{2n+1}\cos(2n+1)\omega_{q}t.$$

The Fourier expansion of the differential charge amplifier output signal is the superposition of sinusoidal signals, and the differential charge amplifier output signal *V_subp_*(*t*) is:

(15) $$y(t)=V_{subp}(t)=\sum_{n=0}^{\infty }b_{n}\sin[(2n+1)\omega_{q}t+\varphi_{n}]+n(t)=v(t)+n(t).$$

where *φ* is the phase difference between *x*(*t*) and *y*(*t*). The useful signal is *v*(*t*) which is interfered with by noise *n*(*t*). *n*(*t*) is the residual display noise and external noise.

The cross-correlation function of *x*(*t*) and *y*(*t*) is [34]:

(16) $$R_{xy}(\tau )=\underset{T\to \infty }{\lim}\frac{1}{2T}\int_{-T}^{T}x(t)y(t+\tau )dt.$$

If the frequencies of *x*(*t*) and *n*(*t*) are different, then *x*(*t*) and *n*(*t*) are independent of each other. In other words, they are not related to each other. The cross-correlation value between *x*(*t*) and *n*(*t*) equals zero. The cross-correlation function *R_xy_*(*τ*) of the two signals *x*(*t*) and *y*(*t*) is:

(17) $$\begin{array}{ll} R_{xy}(\tau ) & =E[x(t)\times y(t+\tau )]\\ & =E[x(t)\times (v(t+\tau )+n(t+\tau ))]\\ & =E[x(t)v(t+\tau )]+E[x(t)n(t+\tau )]\\ & =E[x(t)v(t+\tau )]\\ & =R_{xv}(\tau ) \end{array}$$

The cross-correlation value of two signals disturbed by noise is the same as that of the useful signal. The cross-correlation operation further eliminates the effect of noise on the useful signal and improves the recognition ability of the touch signal.

The integration time of cross-correlation is directly proportional to the frequency difference between two adjacent transmission channels. The integration time is:

(18) $$T=\frac{2\pi }{\omega_{q}-\omega_{q+1}}.$$

where *ω_q_* and *ω_q_*_+1_ are the angular frequency of the *TX_q_* channel and *TX_q_*_+1_ channel, respectively. Then, the cross-correlation value of zero delay between *x*(*t*) and *y*(*t*) is calculated as follows:

(19) $$\begin{array}{ll} R_{xy}(0) & =A\int_{0}^{T}\sum_{n=0}^{\infty }b_{n}\sin[(2n+1)\omega_{q}t+\varphi_{n}]\sum_{n=0}^{\infty }\frac{1}{2n+1}\sin(2n+1)\omega_{q}tdt\\ & =\frac{\pi A}{\omega_{q}-\omega_{q+1}}\sum_{n=0}^{\infty }\frac{b_{n}\cos\varphi_{n}}{2n+1} \end{array}$$

The touch screen equivalent circuit is a band-pass filter. The high harmonic attenuation amplitude is greater than the fundamental frequency signal when transmitting a square wave signal through the touch screen. The high harmonic receiving signal amplitude is much smaller than the fundamental frequency receiving signal amplitude. The cross-correlation value can be approximated as follows:

(20) $$R_{xy}(0)\approx \frac{\pi Ab_{0}}{\omega_{q}-\omega_{q+1}}\cos\varphi_{0}=k_{1}\cos\varphi_{0},$$

where *k*_1_ is a constant, and

(21) $$k_{1}=\frac{\pi Ab_{0}}{\omega_{q}-\omega_{q+1}}.$$

Similarly,

(22) $$R_{x^{\prime }y}(0)\approx k_{1}\sin\varphi .$$

The results of the two cross-correlation operations must not be zero at the same time. The squares of the two cross-correlation results are added to obtain a value independent of the phase difference:

(23) $$R_{xy}{\left(0\right)}^{2}+R_{x^{\prime }y}{\left(0\right)}^{2}=k_{1}{}^{2}.$$

In using this principle, the cross-correlation value independent of the phase difference can be obtained. The weak signal submerged in the display panel noise could be detected by cross-correlation technology.

## 3. The Proposed Low-Computing-Complexity Acquisition Analog Front-End

### 3.1. The Architecture of the Proposed Low-Computing-Complexity AFE

In the mutual capacitive touch system, the AFE transmits a series of orthogonal signals to the touch panel. When there are touch points on the screen, the signals of the corresponding channel received by AFE will change. The touch point coordinates can be obtained by cross-correlation calculation between the changed received signals and the transmitted signals.

**Figure 2:**
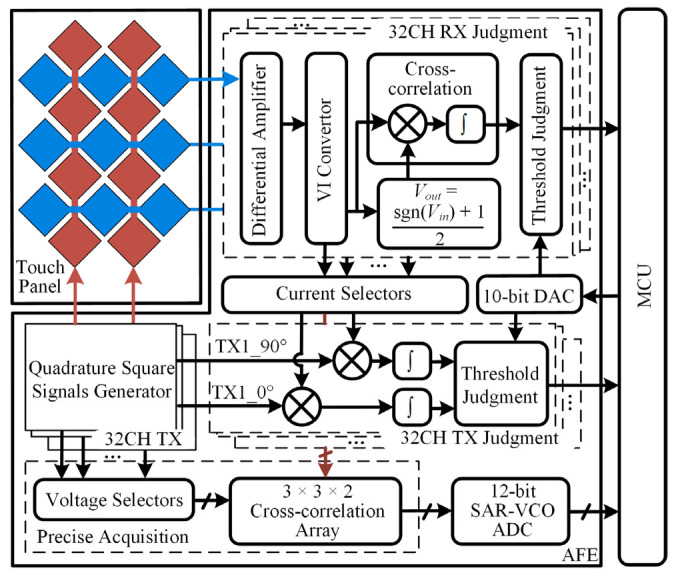
The architecture of the proposed low-computing-complexity AFE circuit based on cross-correlation operation.

The architecture of the proposed low-computing-complexity acquisition AFE circuit is shown in Figure 2. The AFE circuit is composed of TX channels, an RX judgment module, a TX judgment module, a cross-correlation precise acquisition module with a 200 kHz 12-bit SAR-VCO ADC, and three 10-bit digital to analog convertors (DACs). Each TX channel transmits square wave signals with different frequencies to drive the touch panel. At the same time, quadrature square wave signals are generated for the TX judgment module and precise acquisition module. In the RX judgment module, the two adjacent RX signals are subtracted to reduce the influence of display noise by a differential amplifier. This is because the current signal realizes multiplication easier compared with a voltage signal. The voltage to current (VI) converter converts the differential amplifier output voltage signal to a current signal. The current signals are sent into the cross-correlation circuits in the RX judgment module, TX judgment module, and precise acquisition module. The DAC generates three reference voltages for the RX judgment module and TX judgment module. The threshold judgment circuits are provided with a large fault tolerance to avoid the influence of circuit mismatch and residual noise.

**Figure 3:**
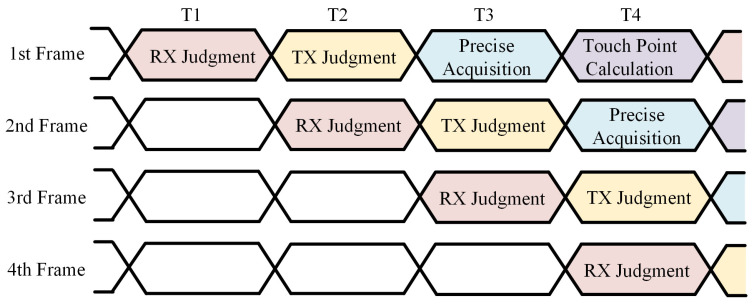
The operation sequence diagram of the proposed low-computing-complexity touch point detection method.

Figure 3 shows the operation sequence diagram of the proposed low-computing-complexity touch point detection method. A pipeline design is adopted to ensure that each module is fully utilized and to improve the frame rate.

The detailed steps of the proposed touch point recognition algorithm are as follows:

1. Step 1: The cross-correlation operation of the *RX* signal and *RX* signal passing through the symbol function realizes the on/off judgment function of the touch point of the RX channel;
2. Step 2: The cross-correlation operations of the *RX* signal with the touch point and each *TX* signal realize the on/off judgment function of the touch point of the TX channel. Rough touch point coordinates are obtained after the above two steps;
3. Step 3: The cross-correlation values around the touch point are sampled precisely by the 12-bit SAR-VCO ADC and sent to MCU;
4. Step 4: The touch point coordinates are accurately calculated with MCU and mapped to display coordinates.

If there are multiple touch points at the same time, repeat steps 2–4 multiple times until all touch points are calculated.

**Figure 4:**
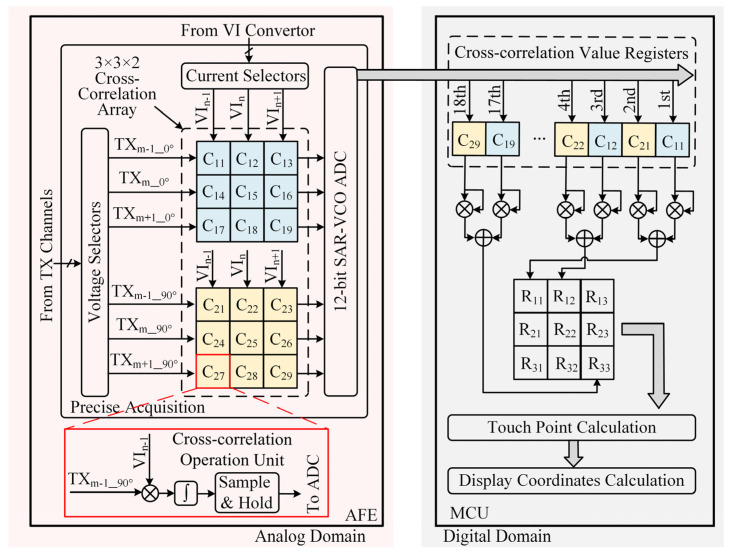
The data flow diagram of the proposed cross-correlation precise acquisition and calculation in the touch system.

Figure 4 is the data flow diagram of the proposed cross-correlation precise acquisition and calculation in the touch system. The cross-correlation operation is realized by the analog circuit in the AFE, and the sum square of two cross-correlation values is realized in MCU. The results of the cross-correlation operation are sent to ADC for acquisition successively.

### 3.2. The Circuit Implementation of the Proposed Cross-Correlation AFE

#### 3.2.1. The RX Judgment Circuit

**Figure 5:**
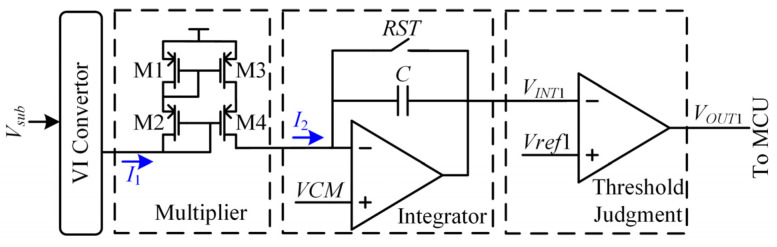
The scheme of the proposed RX judgment circuit.

**Figure 6:**
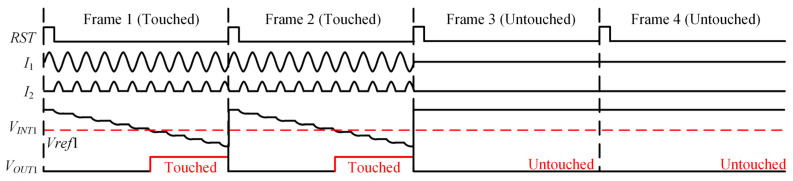
The working sequence of the proposed RX judgment circuit.

Figure 5 and Figure 6 present the scheme and the working sequence of the proposed RX judgment circuit. The cross-correlation circuit and threshold judgment circuit constitute the proposed RX judgment circuit. The cross-correlation circuit is composed of a multiplier and an integrator. 

The voltage to current convertor output current *I*_1_ is:

(24) $$I_{1}=G_{m}V_{sub}.$$

where *G_m_* is the transconductance of the VI convertor. The multiplier is a cascade current mirror. Only parts of *I*_1_ less than zero are copied. The output current *I*_2_ of the multiplier is:

(25) $$I_{2}=-k_{2}I_{1}\cdot \frac{\mathrm{sgn}(-I_{1})+1}{2},$$

where *k*_2_ is the current mirror magnification. If *I*_1_ is greater than zero, *I*_2_ is zero. If *I*_1_ is less than zero, *I*_2_ equals −*k*_2_*I*_1_. The integrator output signal *V_INT_*_1_ is:

(26) $$\begin{array}{ll} V_{INT1} & =VCM-\frac{1}{C}\int_{0}^{T}I_{2}(t)dt\\ & =VCM-\frac{k_{2}G_{m}}{2C}\int_{0}^{T}V_{sub}(t)\times \mathrm{sgn}(V_{sub}(t))dt\\ & =VCM-k_{3}R_{yz}(0) \end{array}$$

where *k*_3_ is a constant and *k*_3_ = *k*_2_*G_m_*/2*C*. *z*(*t*) is the output signal of *V_sub_*(*t*) passing through the symbol function, and *z*(*t*) = sgn[*V_sub_*(*t*)]. *R_yz_*(0) is the cross-correlation function between *V_sub_*(*t*) and *z*(*t*) when the delay time equals zero. The value of cross-correlation must be greater than zero at a time delay of zero if there is a touch point.

The threshold judgment circuit is a comparator that avoids the influence of front-stage circuits mismatch and residual noise. Ideally, if there is not a touch point on the RX channel, the value of *V_INT_*_1_ equals the common voltage. Actually, the value of *V_INT_*_1_ does not equal the common voltage due to the circuits mismatch and noise. It can be concluded that there is no touch point on the RX channel if *V_INT_*_1_ is greater than *Vref*1. If the cross-correlation output in the RX judgment module is less than the threshold voltage *Vref*1, it can be concluded that there is a touch point on the RX channel. The comparator output signal *V_OUT_*_1_ is at a high level when *V_INT_*_1_ is less than threshold voltage *Vref*1.

(27) $$V_{OUT1}=\{\begin{array}{cc} 1, & V_{INT1}<Vref1\\ 0, & V_{INT1}\geq Vref1 \end{array}$$

#### 3.2.2. The TX Judgment Circuit

**Figure 7:**
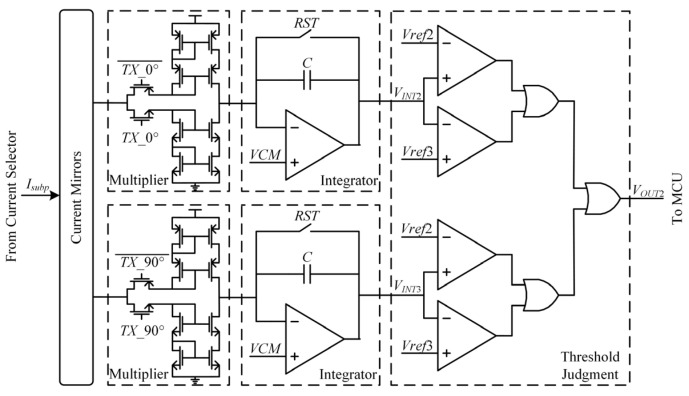
The scheme of the proposed cross-correlation circuit and threshold judgment circuit in the TX judgment circuit.

Figure 7 presents the proposed cross-correlation circuit and threshold judgment circuit in each TX judgment channel. A TX judgment channel is composed of two cross-correlation circuits and a judgment circuit. The selected VI convertor output with touch point performs a cross-correlation operation with *TX* signals of 0° phase and 90° phase. *RST* is a reset signal, and the reset time is set to 10μs. The cross-correlation operation time is adjustable to achieve different frame rates. The value of feedback capacitance *C* in an integrator whose size is 20 × 16 μm is 3.12 pF.

In the multiplier, *TX*_0° and $\bar{TX\_0{}^{\circ}}$ are a pair of square signals whose phases are opposite. If *TX*_0° is high level, the current mirror in the multiplier copies the input current signal *I_subp_*. If *TX*_0° is low level, the current mirror in the multiplier output current equals zero. The multiplication operation is realized by a simple current mirror with a pair of control switches. The integrator output signal *V_INT_*_2_ is:

(28) $$\begin{array}{ll} V_{INT2} & =VCM-\frac{k_{4}}{C}\int_{0}^{T}I_{subp}(t)\times x(t)dt\\ & =VCM-\frac{k_{4}G_{m}}{C}\int_{0}^{T}V_{sub}(t)\times x(t)dt\\ & =VCM-k_{5}R_{yx}(0) \end{array}$$

where *k*_4_ and *k*_5_ are two constants; *k*_4_ is the current mirror magnification of the multiplier and *k*_5_ = *k*_4_*G_m_*/*C*.

Similarly,

(29) $$V_{INT3}=VCM-k_{5}R_{yx^{\prime }}(0).$$

The threshold judgment module is composed of two comparators and three two inputs or gates. The threshold judgment circuit avoids the influence of front-stage circuits mismatch and residual noise. Ideally, if there is not a touch point on the TX channel, the values of *V_INT_*_2_ and *V_INT_*_3_ are equal to the common voltage. Actually, the values of *V_INT_*_2_ and *V_INT_*_3_ do not equal the common voltage due to the circuits mismatch and noise. It can be concluded that there is no touch point on the TX channel if both *V_INT_*_2_ and *V_INT_*_3_ are greater than *Vref*2 and less than *Vref*3. The output signal *V_OUT_*_2_ of the threshold judgment is:

(30) $$V_{OUT2}=\{\begin{array}{cc} 1, & V_{INT2}<Vref2\;||\;V_{INT2}>Vref3\;||\;V_{INT3}<Vref2\;||\;V_{INT3}>Vref3\\ 0, & Vref2\leq V_{INT2}\leq Vref3\;\&\;Vref2\leq V_{INT3}\leq Vref3 \end{array}.$$

It can be concluded that there is a touch point on this TX channel if there is a cross-correlation value less than the threshold voltage *Vref*2 or greater than the threshold voltage *Vref*3.

#### 3.2.3. The Precise Cross-Correlation Sample Circuit

**Figure 8:**
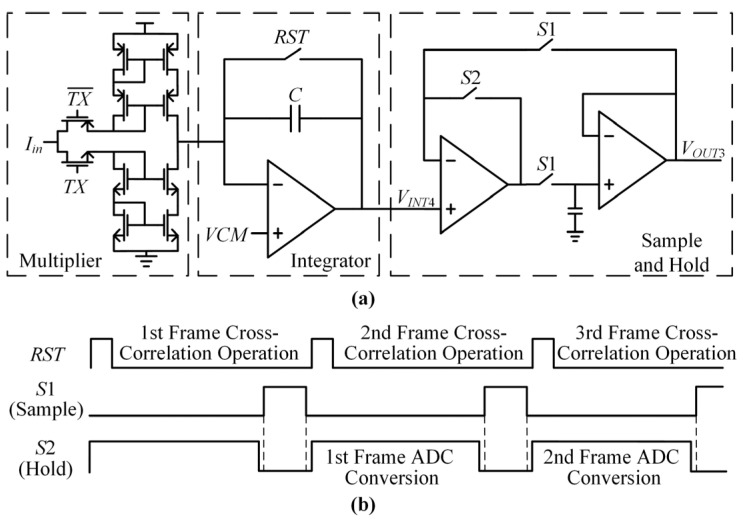
The scheme and working sequence of the proposed cross-correlation circuit, sample, and hold circuit. (**a**) The scheme; (**b**) the working sequence.

Figure 8 presents the scheme of the proposed cross-correlation circuit, sample and hold circuit, and working sequence. Signal *I_in_* is a selected VI convertor output current signal that is the amplified *RX* signal with a touch point. *TX* and $\bar{TX}$ are two opposite square signals selected from TX channels, which are a pair of in-phase signals or quadrature signals. *TX* and $\bar{TX}$ is used to control the opening and closing of the switch to realize the multiplication of *TX* and *I_in_*.

The cross-correlation value is sampled by the sample and hold circuits. *S*1 and *S*2 are two-phase non-overlapping clocks. If *S*1 is high level, the sample and hold circuits work in the sample state. The sample capacitance upper plate voltage equals *V_INT_*_4_. If *S*2 is high level, the sample and hold circuits work in the hold state. The output voltage V*_OUT_*_3_ equals the sample capacitance upper plate voltage. For the next frame of the cross-correlation operations, the cross-correlation values of the previous frame are converted by the 12-bit ADC. The nine data points around the touch point are sampled. Each point contains two cross-correlation values. The 18 cross-correlation values are converted to digital signals by the ADC successively.

The 18 cross-correlation values around the touch point are calculated and fitted with a parabolic equation by MCU. The vertex coordinate of the parabolic equation is the precise touch point coordinate.

## 4. Results and Discussion

**Figure 9:**
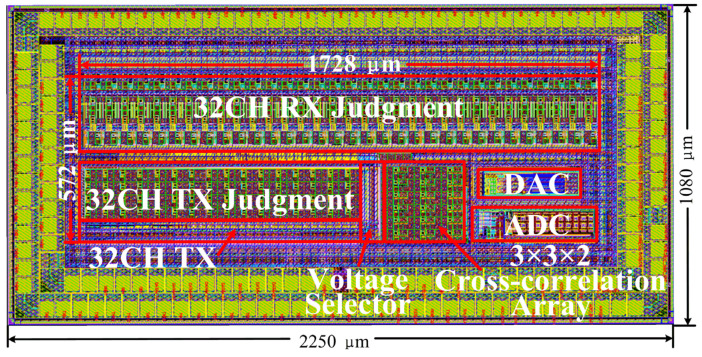
The layout of the proposed low-computing-complexity cross-correlation AFE.

The proposed cross-correlation AFE is designed by the 110 nm CMOS process. Figure 9 shows the layout of the proposed AFE. The total size and active size of the proposed cross-correlation AFE are 2250 × 1080 μm and 1728 × 572 μm, respectively. The average area of the proposed cross-correlation AFE is merely 0.015 mm^2^/channel.

**Figure 10:**
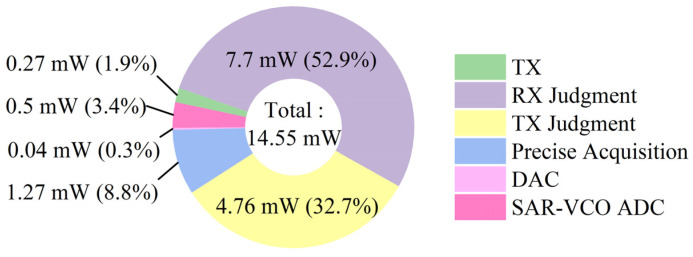
The power consumption of the proposed cross-correlation AFE.

Figure 10 is the power consumption of the proposed cross-correlation AFE. The total power consumption of the proposed cross-correlation AFE is 14.55 mW. There are 32 TX channels and 32 RX channels in the proposed AFE. The total number of channels in the proposed AFE is 64. So, the average channel power consumption is 0.227 mW/channel.

**Figure 11:**
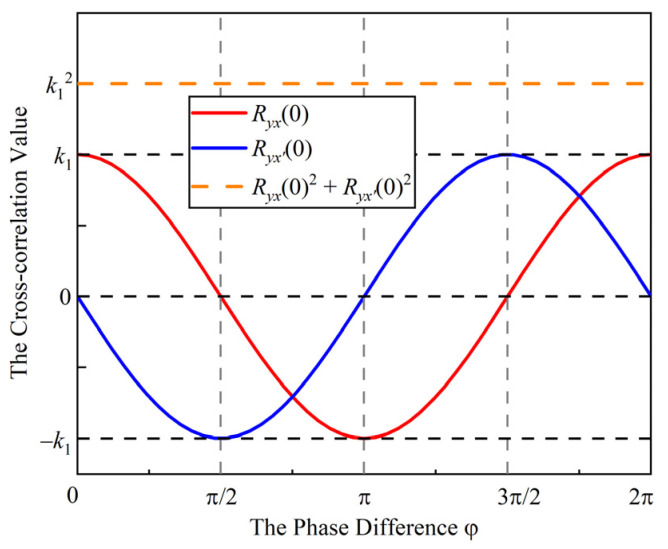
The cross-correlation value variation with the *TX* and subtractor output signal phase difference variation.

Figure 11 shows the cross-correlation value variation with the *TX* and subtractor output signal phase difference variation. If there is a phase difference between the *TX* and subtractor output signal, the cross-correlation value varies with a different phase difference. The sum of squares of the two cross-correlation values is independent of the phase difference.

**Figure 12:**
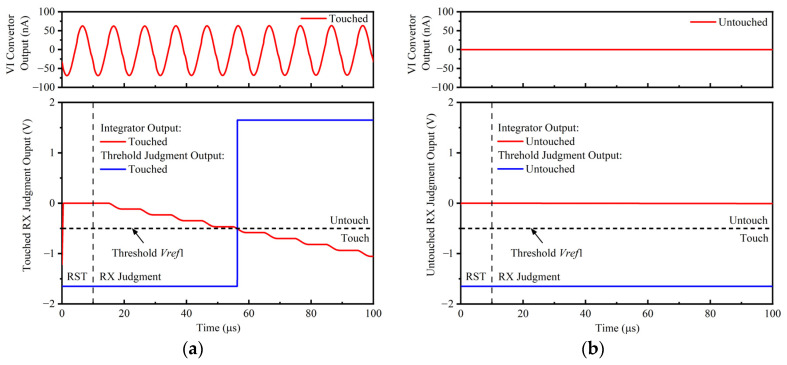
The time-domain waveform of the RX judgment module in the proposed cross-correlation AFE circuit. (**a**) Touched RX judgment output; (**b**) untouched RX judgment output.

**Figure 13:**
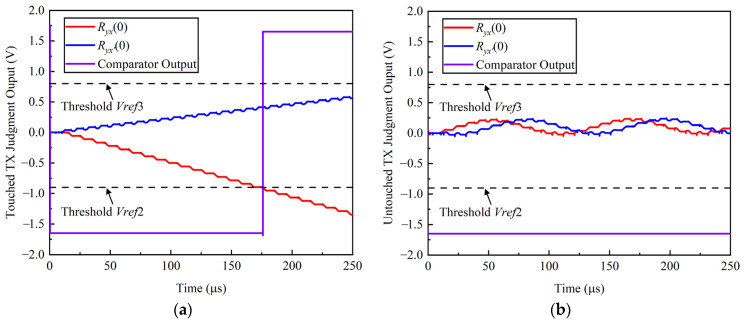
The time-domain waveform of the TX judgment module in the proposed cross-correlation AFE circuit. (**a**) Touched TX judgment output; (**b**) untouched TX judgment output.

Figure 12 and Figure 13 show the time-domain waveform of the RX judgment module and TX judgment module in the proposed cross-correlation AFE circuit, respectively. In Figure 12a, if there is a touch point on the RX channel, the VI convertor output signal is not zero. The cross-correlation calculation result of the VI convertor output signal and symbol function decreases gradually with an increase in integration time. The threshold judgment output jumps to a high level until the cross-correlation result is less than the threshold voltage *Vref*1. In Figure 12b, if there is not a touch point on the RX channel, the VI convertor output signal is zero, the cross-correlation calculation result of the VI convertor output signal and symbol function is zero, and the threshold judgment output is low level all the time. In Figure 13a, if there is a touch point on the TX channel, the cross-correlation calculation result of the VI convertor output signal and *TX* signal decreases or increases gradually with an increase in integration time. The threshold judgment output becomes high level until the cross-correlation result is less than the threshold voltage *Vref*2 or greater than the threshold voltage *Vref*3. In Figure 13b, if there is not a touch point on the TX channel, the cross-correlation calculation result of the VI convertor output signal and *TX* signal oscillates around zero. Moreover, the threshold judgment output is low level all the time.

**Figure 14:**
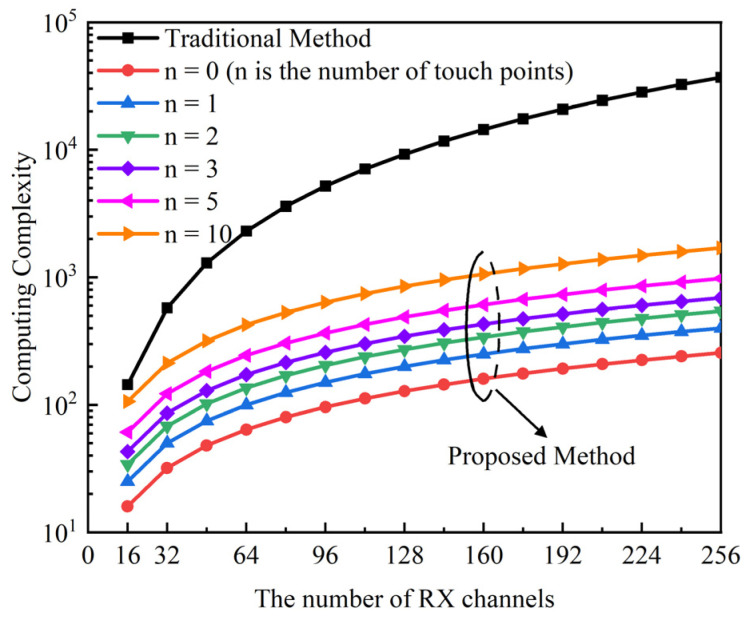
The computing complexity of the traditional method and the proposed method, with the different number of RX channels and the different number of touch points in a frame.

Figure 14 shows the computing complexity of the traditional method and the proposed method, with the different number of RX channels and the different number of touch points in a frame. The ratio of the number of RX channels to the number of TX channels is 16:9. Assuming the number of RX channels and TX channels are *M* and *N*, respectively, the number of touch points is *n*. The computing complexity of the traditional peak search algorithm in MCU is *MN* because a traditional AFE processes the whole panel data. In the proposed cross-correlation AFE, the computing complexity of the RX judgment algorithm is *M*. The single time computing complexity of the TX judgment algorithm is *N*. The biggest computing complexity of the TX judgment algorithm is *nN* if there are *n* touch points. So, the computing complexity of the peak search algorithm in MCU is reduced from *MN* to *M + nN*. Since *n* is much less than *M*, *M + nN* is much less than *MN*. If there are 10 touch points in a frame, the maximum computing complexity of the proposed low-computing-complexity touch point search algorithm is *M* + 10*N*. The ratio of computing complexity of the proposed method versus the traditional method in a frame decreases significantly with an increase in the touch panel size. The power consumption of MCU decreases because of the reduction in computing complexity.

**Figure 15:**
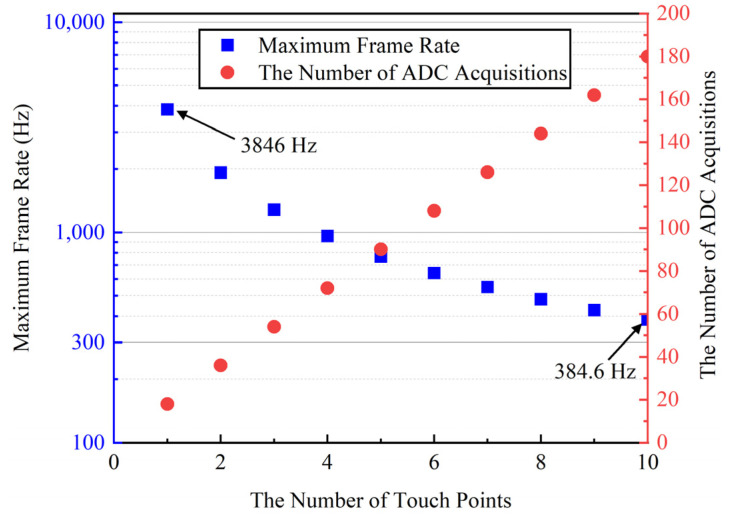
The maximum frame rate and number of ADC acquisitions varies with a different number of touch points.

Figure 15 presents the maximum frame rate and the number of ADC acquisition variations with the different touch points. The frame rate is up to 10 kHz when determining the presence or absence of touch points. The maximum precise acquisition frame rate is 3846 Hz and 384.6 Hz with 1 touch point and 10 touch points, respectively. The frame rate could be set to smaller than the maximum frame rate by adjusting cross-correlation time and reset time to map different devices and screens. We also could set a smaller frame rate to sample more than 10 touch points in a frame. The ADC acquisition number of the traditional method is *MN* in a frame. The ADC acquisition number of the proposed method is 18*n*. If there are 10 touch points in a frame, the maximum ADC acquisition number of the proposed AFE is 180. The ratio of the ADC acquisition number in a frame in the proposed method versus the traditional method decreases significantly with an increase in the touch panel size. The ADC requirement is decreased by the proposed touch signal detection method. There is only an ADC in the proposed AFE. Assuming we adopt the same ADC in the traditional AFE, the number of ADCs is the same as the number of RX channels so as to achieve the same frame rate. There are 32 ADCs in the traditional AFE if the number of RX channels is 32. The power consumption and area of the ADCs in the proposed AFE are 1/32 of the power consumption and area of the ADCs in the traditional AFE. Therefore, the power consumption and die area of the proposed cross-correlation AFE are reduced significantly.

Table 1 provides a simulated performance summary of the proposed cross-correlation AFE in comparison with previous work. Compared with others’ work, the proposed AFE significantly decreases the average single area and power consumption to 0.015 mm^2^ and 0.227 mW, respectively. The signal-to-noise ratio (SNR) of the AFE is defined as follows [16,35]:

(31) $$\mathrm{SNR}=20\log\frac{STouch}{NTouch_{RMS100}},$$

where *STouch* and *NTouch_RMS_*_100_ are defined as follows:

(32) $$STouch=Signal_{Touch,AVG100}-Signal_{Untouch,AVG100}$$

(33) $$NTouch_{RMS100}=\sqrt{\frac{\sum_{n=0}^{99}{\left(Signal_{Touch}[n]-Signal_{Touch,AVG100}\right)}^{2}}{100}},$$

where *Signal_Touch,AVG_*_100_ and *Signal_Untouch,AVG_*_100_ are the average of the 100 cross-correlation values with the touch point and without the touch point, respectively. *RMS100* is the root mean square of 100 data points. The proposed AFE achieves a 46.1 dB SNR with large capacitance and resistance loads for the 65-inch panel.

## 5. Conclusions

In this paper, a touch signal detection method of low computing complexity based on cross-correlation technology for large-size mutual capacitance TSP is proposed. The proposed low computing complexity touch method only samples and calculates the data around the touch points without sampling and calculating the full-screen data to improve the sampling and calculation efficiency. Compared with the traditional method, the proposed touch points search algorithm complexity decreases from *MN* to *M* + *nN*. The maximum ADC acquisition number of the proposed method decreases from *MN* to 18*n*. The touch point search algorithm complexity and ADC acquisition number decrease significantly.

The proposed cross-correlation AFE could detect the weak touch signal submerged in the large display panel noise. The proposed dual cross-correlation acquisition method eliminates the influence of the phase difference between the transmit and receive signals. The proposed AFE reaches 46.1 dB SNR for the 65-inch TSP. The proposed multi-frequency driving AFE circuits distinguish different transmit channels by cross-correlation technology without a complex FFT module for the large mutual capacitance TSP to reduce the die area and power consumption. The average channel area and power consumption of the proposed AFE are reduced to 0.015 mm^2^ and 0.227 mW, respectively. The maximum frame rate reaches 3864 Hz or 386.4 Hz if there is 1 touch point or 10 touch points, respectively. The frame rate is adjustable to map different devices. The proposed cross-correlation AFE achieves a high frame rate without increasing the die area and power consumption.

## Figures and Tables

**Table 1:** Performance summary.

Parameters	JSSC 2019 [12]	CAS-I 2020 [11]	Sensors J. 2019 [32]	JSSC 2021 [24]	Sensors 2020 [14]	This Work
Process	0.18 μm	0.13 μm	0.13 μm	0.18 μm	0.35 μm	0.11 μm
Supply	3.3 V	3 V	3.3/20 V	3.3 V	3.3 V	1.2/3.3 V
Frame Rate	120 Hz	240 Hz	120 Hz	120 Hz	250 Hz	~3846 Hz@ 1 point; ~384.6 Hz@ 10 points
Channels	TX: 36; RX: 64	TX: 31; RX: 15	128 × 72	TX: 8; RX: 17	TX: 15; RX: 12	TX: 32; RX: 32
Total Channels	100	46	200	25	27	62
Power	94.5 mW	11.5 mW	65.31 mW	12 mW	10.4 mW	14.55 mW
Average Power	0.945 mW/channel	0.25 mW/channel	0.327 mW/channel	0.48 mW/channel	0.385 mW/channel	0.227 mW/channel
Active Area	36 mm^2^	1.49 mm^2^	56.25 mm^2^	3.04 mm^2^	4.1 mm^2^	0.988 mm^2^
Average Area	0.36 mm^2^/channel	0.032 mm^2^/channel	0.281 mm^2^/channel	0.122 mm^2^/channel	0.152 mm^2^/channel	0.015 mm^2^/channel
SNR	54 dB	54.2 dB	47.8 dB	37.5 dB	41 dB	46.1 dB
Application	12.2 inch	5.3 inch	32 inch	6.7 inch	N/A	65 inch
ADC Number	32	31	128	1 (shared)	5	1
Computing Complexity	*MN*	*MN*	*MN*	*MN*	*MN*	*M + nN*

## Data Availability

Not applicable.
